# Human Weight Compensation With a Backdrivable Upper-Limb Exoskeleton: Identification and Control

**DOI:** 10.3389/fbioe.2021.796864

**Published:** 2022-01-13

**Authors:** Dorian Verdel, Simon Bastide, Nicolas Vignais, Olivier Bruneau, Bastien Berret

**Affiliations:** ^1^ CIAMS, Sport Sciences Department, Université Paris-Saclay, Orsay, France; ^2^ CIAMS, Université d’Orléans, Orléans, France; ^3^ LURPA, Mechanical Engineering Department, ENS Paris-Saclay, Cachan, France; ^4^ Institut Universitaire de France, Paris, France

**Keywords:** weight support, rehabilitation robotics, joints misalignments, feed-forward control, human parameters identification, human/exoskeleton interaction

## Abstract

Active exoskeletons are promising devices for improving rehabilitation procedures in patients and preventing musculoskeletal disorders in workers. In particular, exoskeletons implementing human limb’s weight support are interesting to restore some mobility in patients with muscle weakness and help in occupational load carrying tasks. The present study aims at improving weight support of the upper limb by providing a weight model considering joint misalignments and a control law including feedforward terms learned from a prior population-based analysis. Three experiments, for design and validation purposes, are conducted on a total of 65 participants who performed posture maintenance and elbow flexion/extension movements. The introduction of joint misalignments in the weight support model significantly reduced the model errors, in terms of weight estimation, and enhanced the estimation reliability. The introduced control architecture reduced model tracking errors regardless of the condition. Weight support significantly decreased the activity of antigravity muscles, as expected, but increased the activity of elbow extensors because gravity is usually exploited by humans to accelerate a limb downwards. These findings suggest that an adaptive weight support controller could be envisioned to further minimize human effort in certain applications.

## 1 Introduction

Active exoskeletons are promising devices in many areas and their potential benefits in various applications have been extensively studied in the past decades. In particular, active exoskeletons have been tested as a solution to improve rehabilitation processes in stroke patients (Frisoli et al., 2007; Pons, 2010; Frisoli et al., 2012; Tan et al., 2020). They are expected to help patients to recover more quickly and more permanently their motor functions due to the advantage offered by robotics such as reliability and repeatability (Huang and Krakauer, 2009). Because of their versatility, active exoskeletons can even be used to help disabled people to perform daily living activities such as walking (Benabid et al., 2012; Mooney et al., 2014; Huo et al., 2019). Furthermore, benefits in terms of reducing fatigue (Mooney et al., 2014; Young et al., 2017) and preventing musculoskeletal disorders at work are expected (Sylla et al., 2014; Bogue, 2015; de Looze et al., 2016). All these applications are conditioned by several critical functionalities including transparency (Ajoudani et al., 2018) and the ability to compensate for the user’s weight (Just et al., 2020). Transparency is the ability of an exoskeleton to influence human movement as little as possible when worn (Proietti et al., 2016) and has already been extensively studied (Jarrasse et al., 2008; Jarrasse and Morel, 2012; Pirondini et al., 2016; Bastide et al., 2018; Verdel et al., 2021b). It is for instance critical at the end of a rehabilitation process to ensure that the robot will not lead the user to adopt abnormal motor patterns. Furthermore, transparency is a prerequisite for successful integration in a workstation where the worker does not need permanent assistance. Weight support (WS) has received less attention but can be defined as the fact of compensating both the exoskeleton dynamics (i.e., being transparent) and the weight of the human body segments. Having a robot carrying the human segments is useful to relieve the user from the strong constraints and efforts imposed by gravity and to ease its movements. This function can be very helpful for patients with muscle weakness and critical to rehabilitation protocols as it can substantially increase their motor repertoire (Prange et al., 2006; Frisoli et al., 2007; Frisoli et al., 2012; Just et al., 2020). We note that WS has also been used in lifting and carrying tasks in the industrial context in order to decrease the occurrence of musculoskeletal disorders in workers (Theurel et al., 2018; Treussart et al., 2020). In any case, the goal is to reduce the user’s muscular efforts related to gravity. Besides studies restricted to the horizontal plane (Tyryshkin et al., 2014; Otaka et al., 2015; Mochizuki et al., 2019), control strategies for WS in general movements and their impact on the human motion have been rarely studied for the upper limb, except in the case of the hand (Carson et al., 2009; Oytam et al., 2010) (see related works in Section 1.2 for details). Indeed, active WS requires building a weight compensation model that includes the estimation of various parameters such as segment mass and location of its center of gravity, which is a long-standing anthropometric issue (Hatze, 2005) (see related works in Section 1.1 for details). Therefore, designing accurate models and protocols for the *in situ* identification of human masses is the first step towards achieving generic WS. Designing efficient control laws is the second step to track the identified weight model during dynamic movements. These two steps are crucial towards an easier integration of exoskeletons with effective WS functionality.

### 1.1 Human Segments Identification

In the seminal work of Chandler et al, the inertial properties of human body segments was estimated from six male cadavers (mean age 54). These data led to the creation of anthropometric tables such as De Leva tables (de Leva, 1996a; de Leva, 1996b) and Winter tables (Winter, 1990). Nevertheless, these tables are notoriously inaccurate and are not adapted to design a personalized compensation. Other methods such as X-rays and tomography analysis have led to better results (Hatze, 2005) but are not satisfying in terms of material as they require heavy devices irradiating the participants. Non-invasive approaches based on motion capture analysis emerged with this technology, allowing precise identification of body geometry (Todorov, 2007). Motion capture-based identification techniques were developed to provide results on inertial parameters (Venture et al., 2008; Jovic et al., 2016), viscoelastic parameters (Venture et al., 2007) and can be applied in real-time (Venture et al., 2009; Ayusawa et al., 2011) during optimal movements in the identification sense (Bonnet et al., 2016). Most of these experiments were conducted on relaxed (i.e., passive) participants, which was verified by means of electromyographic (EMG) measures. All these techniques still present the major disadvantage of requiring a special room equipped with motion capture technology, which few hospitals and companies can afford. An alternative way to identify human parameters would be an *in situ* identification using exoskeleton data (such as kinematic or force/torque (FT) measures) and, therefore, avoiding the use of external measurement tools. Fewer studies have been conducted in this perspective. The upper-limb masses identification was carried out with different methods (Just et al., 2017; Just et al., 2020) suggesting that the most efficient method is to identify the vertical force generated by the segment when the human is completely relaxed, as during certain experiments based on motion capture techniques. This idea of *in situ* identification was also developed for lower limbs (Hwang and Jeon, 2015). The force measurement is, in these experiments, either obtained from FT sensors placed at the level of the connection between the human and the robot or from torque sensors placed at the joints of the robot. The main limitation of these studies was the fact that joint misalignments (JM) between the user and the exoskeleton were not considered. Indeed, these misalignments have an impact on the interaction efforts between the user and the exoskeleton (Jarrasse and Morel, 2012). Therefore, they can lead to under or over compensation of weight because of an erroneous identification of the human segment mass. As a consequence, the present paper focuses on introducing a general model of weight taking into account JM for a limb with the objective to generate more accurate compensations.

### 1.2 Weight Model Tracking

The second step to achieve an accurate WS is to carefully follow the identified weight model. As previously introduced, WS is a necessary step in many functional rehabilitation processes (Prange et al., 2006; Frisoli et al., 2007; Frisoli et al., 2012). Therefore, its effect have been studied on disabled patients and healthy participants often using passive systems (Prange et al., 2009b; Perry et al., 2017; Puchinger et al., 2018; Hull et al., 2020; Perry et al., 2021). These investigations were oriented towards the analysis of muscle activities and workspace in different configurations. In the horizontal plane (F. Beer et al., 2007), in the sagittal plane (Prange et al., 2009a; Prange et al., 2009b; Perry et al., 2021) and during three-dimensional movements (Coscia et al., 2014; Puchinger et al., 2018; Runnalls et al., 2019; Hull et al., 2020), a global decrease of electromyographic (EMG) activities and an increase in the number of muscular synergies were observed. Moreover, an increase in the workspace of disabled patient was also reported. Nevertheless, these findings did not address the problem of compensating weight with an active device in the vertical plane. In fact, few studies considered this problem. As previously mentioned, it was discussed for rhythmic hand movements (Carson et al., 2009; Oytam et al., 2010) using box Jenkins models for motors in order to analyze the stability of human movements under different conditions. Some studies about upper-limb WS with active exoskeletons such as (Just et al., 2017; Just et al., 2020) described different compensation methods but did not focus on the associated design of an accurate control law. Furthermore, these studies either considered lightweight limbs as hands or dealt with movements performed in horizontal plane that induce a constant weight torque. WS could even be obtained natively in the horizontal plane without dedicated control with the KinArm exoskeleton (Tyryshkin et al., 2014; Otaka et al., 2015; Mochizuki et al., 2019). In these cases, only a partial movement set generating few variations in weight torque is considered, which does not induce the problems that occur in more general settings. As a consequence, the present paper will focus on problem of WS for an arm moving in the sagittal plane as this will generate substantial variations in gravitational torques. The objectives are thus to 1) build an accurate weight compensation model *in situ* for each participant considering JM and 2) design a WS control law that follows this model despite variations of gravitational torques during motion in the vertical plane.

**FIGURE 1:**
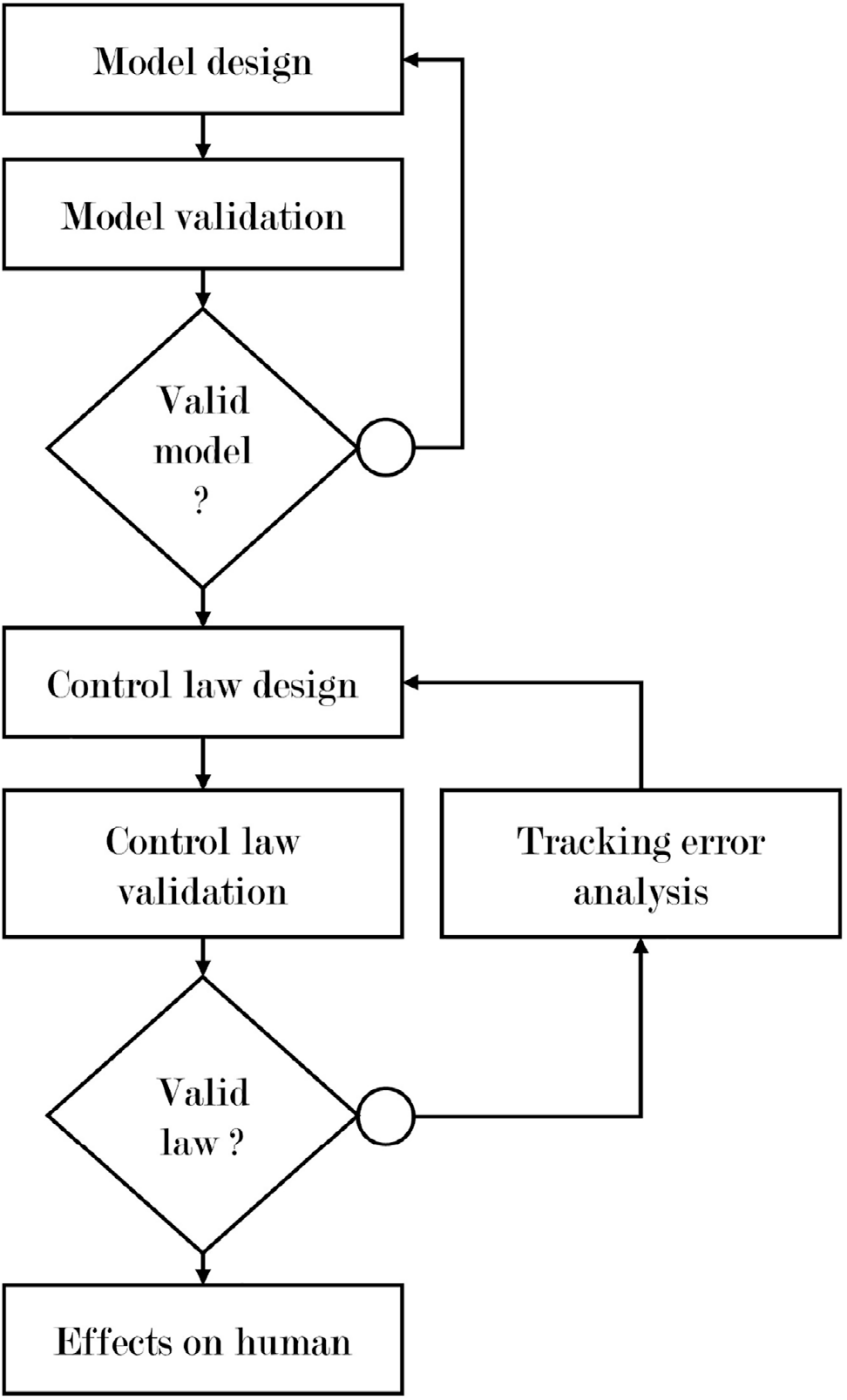
Applied methodology.

Considering these two main objectives, the general methodology applied in the present study is presented in Figure 1, and the rest of this paper is organized as follows. In Section 2.1 a weight model and its identification procedure are presented. Then, in Section 2.2, the construction of the weight compensation control law is described and some successive solutions are proposed. In Section 2.3 a validation task allowing to compare the different solutions and validate the control law’s design methodology is described. All the experimental results are presented in Section 3 and discussed in Section 4.

## 2 Materials and Methods

### 2.1 Weight Model With Joint Misalignments and Identification

#### 2.1.1 General Case

**FIGURE 2:**
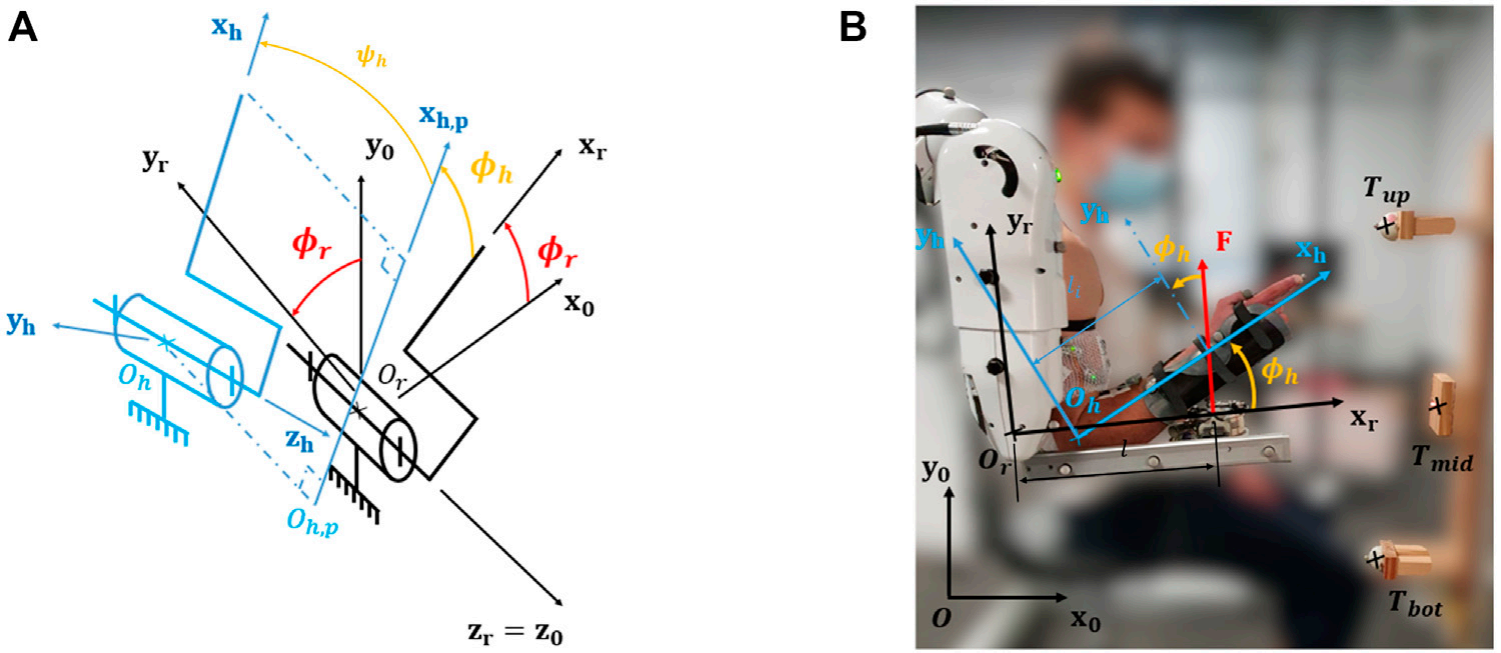
Misalignments between human and exoskeleton. **(A)** General situation. **(B)** Sagittal plane case study.

The first step to build a WS control is to identify precisely the mass of the limb used in the movement. Given the fact that JM between the exoskeleton and the user are inevitable (Jarrasse and Morel, 2012), these misalignments must be identified to build a homogeneous weight compensation. This has not yet been explored in previous weight compensation studies as the assumption of perfect alignment between the user and the exoskeleton is often implicitly made. In the general case, misalignments between a human segment and an exoskeleton link can be described by three rotations and three translations as presented in Figure 2A.

In the case of spatial motions, the first step of the proposed model is to define a set of vertical planes containing the robot segments according to ***ϕ***_***r***_ (the joint coordinate vector of the robot). The successive projections of the human limb in these different planes make it possible to define a vector ***ϕ***_***h***_(***ϕ***_***r***_) containing the different JM to be identified. Then, if the robot is composed of revolute joints, which is commonly the case in upper-limb exoskeletons, e.g., see (Frisoli et al., 2007; Garrec et al., 2008; Gopura et al., 2016; Brygo et al., 2017; Ercolini et al., 2018), a last transformation *R*_*rh*,*i*_(**x**_**ri**_, ***ϕ***_***r***_) (i.e., a rotation depending on ***ϕ***_***r***_ around the axis **x**_**ri**_ which supports the *i*th segment) must be applied to compute the component of the human weight torque that is kinematically compatible with the *i*th joint. The detailed description of the proposed model is available in Supplementary Appendix A1.1 for one human and one robot limb. It should be noted that the identification process also requires appropriate FT sensors.

As this reduction of the problem to planar problems is always possible by projecting **x**_**h**_ (i.e., the vector supporting the human limb as described in Figure 2A) in the defined vertical planes, the rest of the present study will focus on planar human movements in the sagittal plane. More precisely, we will focus on accurately identifying and compensating the weight of a human forearm and hand in the sagittal plane during goal-directed movements.

#### 2.1.2 Sagittal Plane Case Study

In the present study, a modified ABLE exoskeleton is used (see Section 2.3.1 for detailed presentation). This exoskeleton is modified to be as compliant as possible in terms of connection to the user. More specifically, residual torques and forces due to position and orientation errors between the exoskeleton and the user are removed by a ball joint coupled to a prismatic joint (i.e., a slider on a rail), which aims at improving the quality of the interaction in terms of comfort. These modifications imply that measures of the FT sensor along **x**_**r**_ are not exploitable. Therefore, our WS control can only rely on the local normal measure of the sensor along **y**_**r**_ in this case study which is a slight difference with the method proposed in (Just et al., 2020). In this situation, the rotation previously defined by *ϕ*_*r*_ is directly equal to the rotation induced by a robot motor, therefore we have *ϕ*_*r*_ = *q*, where *q* is the angular position of the robot elbow. The weight model (i.e., the evolution of the weight torque *τ*_*h*_ with regard to *q*, the angular position of the robot with a horizontal reference) that should be identified is a particular case of Eq. 18 and is described in Equation 1,

$$\begin{array}{ll} \tau_{h} & =\left(x_{g}x_{h}\times W_{h}\right)\cdot z_{0}\\ & =-m_{f,h}gx_{g}\cos\left(q+\phi_{h}\left(q\right)\right) \end{array}$$ (1)

where *τ*_*h*_ is computed under the assumption that the center of mass of the human segment is on the axis (*O*_*h*_, **x**_**h**_) (as in (Just et al., 2020)), *m*_*f*,*h*_ is the addition of the masses of the forearm, the hand of the user and the connection to the robot that must be identified, *x*_*g*_ is the center of mass position in the human frame, *q* is the angular position of the robot elbow (measured by incremental encoders) and *ϕ*_*h*_(*q*) is the misalignment between the robot and the user forearm segments that must be identified. The resulting force interaction model that must be applied by the robot to compensate for the weight is obtained through equilibrium of torques and described in Eq. 2 by

$$F=m_{f,h}\frac{x_{g}}{l_{i}}g\frac{\cos\left(q+\phi_{h}\left(q\right)\right)}{\cos\left(\phi_{h}\left(q\right)\right)}$$ (2)

where *F* is the opposite of the normal interaction force measured by the FT sensor and *l*_*i*_ is the distance between the human elbow and the interaction point along **x**_**h**_. As the physical interface with the robot completely envelopes the human forearm, the interaction point is modeled as a point belonging to the axis (*O*_*h*_, **x**_**h**_). Note that this interaction force model is only valid for static situations where inertial and viscous terms and human muscles activation does not exist or is negligible. As a consequence, this model can only be identified on static positions of the robot and with a relaxed user as it is often the case when trying to compensate for weight (Hwang and Jeon, 2015; Just et al., 2017; Just et al., 2020). It is interesting to note that using a force equilibrium seems to be inappropriate in this situation. Indeed, it would require to neglect the interactions at the level of the human joint to solve the problem, which amounts to use a classical weight model without JM.

By design, the torque at the robot elbow joint resulting from this weight compensation model is equal to the interaction force of Eq. 2 multiplied by the lever arm between the robot elbow joint center *O*_*r*_ and the direction of this force, which corresponds to the distance between *O*_*r*_ and the slider (see Figure 2B). This is described by Equation 3,

$$\begin{array}{ll} \tau_{i} & =lF\\ & =lm_{f,h}\frac{x_{g}}{l_{i}}g\frac{\cos\left(q+\phi_{h}\left(q\right)\right)}{\cos\left(\phi_{h}\left(q\right)\right)} \end{array}$$ (3)

where *τ*_*i*_ is the resulting interaction torque to apply for weight compensation and *l* is the distance between *O*_*r*_ and the slider.

#### 2.1.3 Model Without JM

For comparison purposes, the model without JM is defined below. As JM are described by *ϕ*_*h*_ in our framework, the case *ϕ*_*h*_ ≜ 0 corresponds to a model without JM and is described by Equation 4,

$$\tau_{i,wjm}=lm_{f,h}\frac{x_{g}}{l_{i}}g\cos\left(q\right)$$ (4)

where *τ*_*i*,*wjm*_ is the torque to apply to compensate for weight without JM.

#### 2.1.4 Optimization Problem

The one degree of freedom weight model presented in Eq. 2 can be adapted to formulate an optimization problem in the least squares sense. In the rest of the present study, only a zero-order approximation of *ϕ*_*h*_(*q*) (i.e., *ϕ*_*h*_(*q*) ≈ *ϕ*_*h*_) is considered as experiments tends to show that this approximation is reasonable on our tested movement ranges (see Section 3.1). This approximation might not hold for movements of large amplitude (typically for *ϕ*_*h*_ + *q* > 40° and *ϕ*_*h*_ + *q* < −60° in our case). This is not a problem for the future generalization as a more complex model of *ϕ*_*h*_(*q*) could be identified through experiment. The resulting optimization problem is presented in Equation 5,

$$\left(\begin{array}{c} {\hat{m}}_{eq,h}\\ {\hat{\phi }}_{h} \end{array}\right)={\mathrm{argmin}}_{m_{eq,h},\phi_{h}}{\left(F-m_{eq,h}g\frac{\cos\left(q+\phi_{h}\right)}{\cos\left(\phi_{h}\right)}\right)}^{2}$$ (5)

where ${\hat{m}}_{eq,h}$ and $\hat{\phi_{h}}$ are the estimated values of *m*_*eq*,*h*_ and *ϕ*_*h*_, respectively, and *m*_*eq*,*h*_ = *m*_*f*,*h*_ *x*_*g*_/*l*_*i*_. In order to accurately estimate these two values, as many measures of *F* and *q* as possible must be collected for each participant during a static and passive experiment. Therefore, the identification procedure detailed below is carried out.

#### 2.1.5 Participants

The evaluation of the present approach was performed with 17 healthy right-handed participants. Basic anthropometric characteristics of the participants were as follows: 7 females, 10 males, mean weight 70.7 ± 9.7 kg, mean height 176 ± 7.8 cm, mean age 24 ± 4.9 years old, mean forearm length 25.5 ± 1.4 cm and mean hand length 18.6 ± 1.3 cm. It should be noted that limb lengths were measured approximately with a measuring tape. Therefore, results in terms of limb length are not exploitable as relevant anthropometric data but still allow us to have a first approximation of the population on which the control laws have been tested. A written informed consent was given by each participant as required by Helsinki declaration (World Medical Association, 2001). The experimental protocol was approved by the ethical committee for research (Université Paris-Saclay, 2017-34). The written consent and the approval of the protocol were also obtained for all the experiments presented in the rest of the present paper.

#### 2.1.6 Identification Protocol

As stated previously, the identification of the human arm’s parameters is performed under static conditions and with the participant being as passive as possible. Participants were placed with their shoulder at the intersection of the first three joints of ABLE. They were connected to ABLE at the level of their forearm and arm (see Section 2.3 for details). Participants were asked to relax while the elbow joint of the robot moved slowly between 20 positions equally spread across its whole work-space: $\left[-85.94{}^{\circ},39.53{}^{\circ}\right]$ . Each position was statically maintained during 5 s. Measures from electromyographic (EMG) sensors were used to check in real-time that the participant was relaxed. Angular positions of the robot and interaction forces were measured at 1 kHz. The resolution of the optimization problem presented in Eq. 5 was then carried out using the “lsqnonlin” function from the “Optimization Toolbox” of Matlab^©^. For comparison purposes, the same optimization was carried out with *ϕ_h_* ≜ 0 (only the observed mass was identified in this case).

After identification of the model, the mean absolute error (MAE) between predictions and measures was computed for each subject and both models (with and without JM) as expressed in Equation 6

$$MAE=\frac{1}{N}\sum |\hat{F}\left(q,\phi_{h}\right)-F|$$ (6)

where *N* is the number of samples, $\hat{F}\left(q,\phi_{h}\right)$ is the predicted force resulting from model identification and *F* is the measured force.

A pairwise *t*-test comparison was then conducted between the two sets of errors using the Pingouin package (Vallat, 2018). The significance level was fixed at *p* < 0.05.

#### 2.1.7 Adjustable Compensation

The WS level can be adjusted easily by adjusting the model presented in Eq. 3. Indeed, adding a real coefficient to the weight model allows to choose directly the level of the desired compensation. The modified model is expressed in Equation 7,

$$\tau_{i}=\alpha lm_{eq,h}g\frac{\cos\left(q+\phi_{h}\right)}{\cos\left(\phi_{h}\right)}$$ (7)

where *α* is the introduced coefficient. For example, during the course of rehabilitation, *α* can be varied between 1 (for a complete WS) and 0 (for a transparent behavior at the end of a therapy). It should be noted that the human parameters must be identified before changing the compensation level.

Achieving an effective WS requires the ability to accurately track the model defined in Eq. 3, which will be the main focus of the rest of Section 2.

### 2.2 Weight Support Control Laws

This section describes the off-line construction of a general WS control law based on compensation errors observed during experiments. In other terms, the underlying learning process necessary to design the control laws is described in the current section.

#### 2.2.1 Initial Force Feedback Control Law

All the control laws used in the present study are based on an accurate compensation of the robot dynamics. This compensation is based on the methods described in (Verdel et al., 2021b). Over this dynamic compensation of the robot, the FT sensor is used to build a feedback control based on the normal interaction forces with the user (Verdel et al., 2021a). In classical approaches, the control is often assured by a partial or complete Proportional Integral Derivative (PID) corrector. The parameters of this type of corrector are system-dependent and task-dependent. In our case a PI corrector was implemented as the derivative coefficient generated instabilities. The control law resulting from the classical PI approach is described by Equation 8,

$$\tau_{ctrl}=K_{p}\left({\hat{\tau }}_{i}\left(q,\phi_{h}\right)-\tau_{i}\right)+K_{i}\int_{0}^{t}\left({\hat{\tau }}_{i}\left(q,\phi_{h}\right)-\tau_{i}\right)dt+{\hat{\tau }}_{comp}\left(\ddot{q},\dot{q},q\right)$$ (8)

where *τ*_*ctrl*_ is the control torque to apply at the elbow joint of the robot, ${\hat{\tau }}_{i}\left(q,\phi_{h}\right)$ is the estimated WS control torque resulting from Eq. 3, *τ*_*i*_ is the measured interaction torque resulting from FT sensor measurements and ${\hat{\tau }}_{comp}\left(\ddot{q},\dot{q},q\right)$ is the estimated compensation of the robot dynamics based on the model presented in (Verdel et al., 2021b) and in Eq. 9. This model is dependent on $\ddot{q}$ (respectively $\dot{q}$ and *q*) the angular acceleration (respectively angular velocity and position) of the elbow joint. The angular velocity and acceleration are estimated by numerical differentiation of the angular positions of the robot. The angular acceleration thereby estimated is too noisy to be used in the exoskeleton control, therefore, inertial terms are not taken into consideration in the dynamic compensation. The control scheme describing this behavior is presented in Figure 4 in black. This law will be called BC (for Basic Compensation) in the rest of the present paper. The compensation of robot dynamics is described by Equation 9,

$${\hat{\tau }}_{comp}\left(\ddot{q},\dot{q},q\right)=\hat{M}\left(q\right)\ddot{q}+\hat{C}\left(q,\dot{q}\right)+\hat{G}\left(q\right)+\mathrm{sign}\left(\dot{q}\right){\hat{\tau }}_{C}+\hat{\nu }\dot{q}$$ (9)

where $\hat{M}\left(q\right)$ is the estimated inertia of the segment, $\hat{C}\left(q,\dot{q}\right)$ is the estimated Coriolis/centrifugal efforts, $\hat{G}\left(q\right)$ is the estimated gravitational torque induced by the robot weight, ${\hat{\tau }}_{C}$ is the estimated Coulomb dry friction torque, $\hat{\nu }$ is the estimated viscous friction coefficient and ${\hat{\tau }}_{comp}$ is the estimated motor torque of the robot used to compensate for the robot dynamics.

#### 2.2.2 Feedforward Term Dependent on Theoretical Interaction Force

**FIGURE 3:**
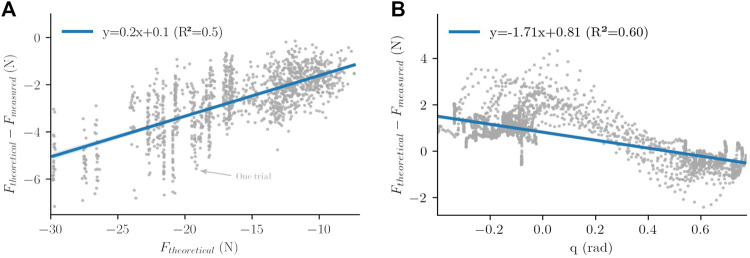
Mean linear models extracted from experiments. **(A)** Interaction force versus theoretical force and mean linear behavior. **(B)** Interaction force versus angular position and mean linear behavior.

As it will be seen in the results, the initial force-feedback control law was insufficient to ensure an efficient tracking of the theoretical model during vertical arm movements (see Section 3.2 for details). The analysis of measured interaction forces during experiments with this classical approach allowed us to uncover that model tracking errors depended on the magnitude of the theoretical force with an affine trend (see Figure 3A).

**FIGURE 4:**
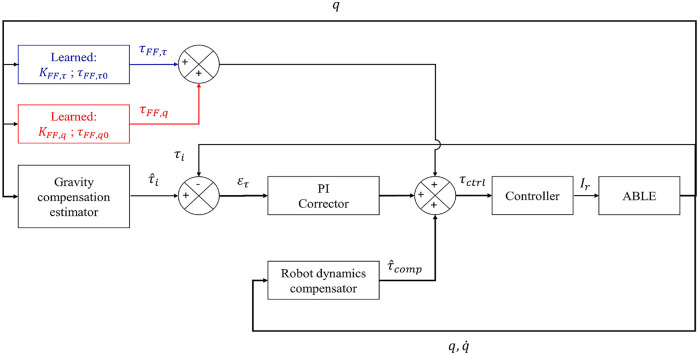
Control schemes used during the present study. Black: basic PI control loop and weight torque estimation. Blue: FFTF term. Red: FFCM term.

This trend revealed by the experiments allows to add a feedforward term to the previous control law, based on a linear model extracted from errors analysis. This term is defined by Eq. 10 as follows:

$$\tau_{FF,\tau }=K_{FF,\tau }{\hat{\tau }}_{i}\left(q,\phi_{h}\right)+\tau_{FF,\tau 0}$$ (10)

where *τ*_*FF*,*τ*_ is the feedforward torque added to the control law presented in Eq. 8, *K*_*FF*,*τ*_ is the slope of the identified linear model of Figure 3A, ${\hat{\tau }}_{i}\left(q,\phi_{h}\right)$ is the theoretical torque resulting from Eq. 1 and *τ*_*FF*,*τ*0_ is the *Y*-intercept of the identified linear model of Figure 3A multiplied by *l*. The approach consists in learning the error behavior across trials and participants to improve the overall WS quality, by adding this linear model as a feedforward torque. This learned model is represented in blue in Figure 4 and in Eq. 12. The law taking into account this model will be called FFTF (for Feedforward Force based on Theoretical Force) in the rest of the present paper.

#### 2.2.3 Feedforward Term Dependent on the Robot Angular Position

Despite significant improvements in terms of tracking error, it will be seen that the previous feedforward approach still generated too much tracking errors and was still inadequate to ensure an efficient tracking of the theoretical model (see Section 3.2 for details). The analysis of measured interaction forces during experiments on the previous approach allowed us to exhibit another mean behavior of the compensation errors presented in Figure 3B, following the learning approach introduced previously.

The behavior exhibited by the experiments allows to complete our feedforward approach by adding another linear compensation depending on the angular position of the robot. This model is described by Equation 11,

$$\tau_{FF,q}=K_{FF,q}q+\tau_{FF,q0}$$ (11)

where *τ*_*FF*,*q*_ is the second feedforward torque added to the control law presented in Eq. 8, *K*_*FF*,*q*_ is the slope of the identified linear model of Figure 3B, *q* is the angular position of the robot and *τ*_*FF*,*q*__0_ is the *Y*-intercept of the identified linear model illustrated in Figure 3B. The measures given in Figure 3B do not suggest the use of a linear model at first sight. Nevertheless, for point-to-point movements between various targets, their pseudo-sinusoidal form is mainly due to delay and lack of inertia compensation in the control laws. These errors could be compensated with intention detection, but this is not the focus of the present study. The learned model is represented in red in Figure 4 and in Equation 12). This law will be called FFCM (for Feedforward Force Complete Model) in the rest of the present paper. The complete WS control law thereby constructed is described by Equation 12,

$$\begin{array}{ll} \tau_{ctrl} & =K_{p}\left({\hat{\tau }}_{i}\left(q,\phi_{h}\right)-\tau_{i}\right)\\ & +K_{i}\int_{0}^{t}\left({\hat{\tau }}_{i}\left(q,\phi_{h}\right)-\tau_{i}\right)dt+{\hat{\tau }}_{comp}\left(\dot{q},q\right)\\ & +\tau_{FF,\tau }+\tau_{FF,q} \end{array}$$ (12)

#### 2.2.4 Individualized Compensation

It should be noted here that in a rehabilitation or industrial context, it would be possible to learn the FFTF and FFCM control laws for only one individual and, therefore, build a fully personalized control. Indeed, the process of learning errors requires an amount of data that can be obtained on only one individual. As different levels of theoretical forces are needed, changing the level of applied WS (by modifying *α* from Eq. 7, in Section 2.1) is a solution to identify the parameters of the FFTF law. The identification of the parameters of a personalized FFCM would also be possible as it would only require to perform a small number of upward and downward movements on a large amplitude (such as 60°). Personalized learning could also be performed on-line contrary to our general learning that requires to gather data from different participants.

#### 2.2.5 Control Modes

Two control modes are used in our experiments, the first one is a “transparent” control. In this mode, the control is designed to minimize the normal interaction force (i.e., minimize *F*). This “transparent” control mode is used as a baseline condition to quantify alterations in the human movement when submitted to different WS controls.

The second control mode corresponds to a classical WS where the robot should compensate its own dynamics and the weight of the forearm/hand of the user during movements in the sagittal plane. Therefore, the robot is controlled to maintain a normal interaction force that follows the model presented in Eq. 1. The three previous control laws (i.e., BC, FFTF and FFCM) are tested on this control mode and their respective capacities in terms of model tracking are tested and compared in Section 3.2.

### 2.3 Design of the Experimental Validation of the Control Laws

#### 2.3.1 Materials and Task

##### Participants

As described in Section 2.2, three control laws are tested sequentially. The second control law (FFTF) was constructed from data recorded with the first law (BC) and the third control law (FFCM) was constructed with data recorded when using the second control law (FFTF). Therefore, three sessions were necessary to build and test our three control laws. Data relative to the participants of each session are reported in Tables 1, 2.

**TABLE 1:** Data relative to the participants: *N* is the number of participants, FL is the forearm length and HL is the hand length.

Session	N	Sex	Age (years)	Weight (kg)	Height (cm)	FL (cm)	HL (cm)
1	29	10 F; 19 M	23.2 ± 3.0	67.1 ± 11.8	174.7 ± 7.6	25.5 ± 1.6	18.8 ± 1.0
2	17	7 F; 10 M	24 ± 4.9	70.7 ± 9.7	176 ± 7.8	25.5 ± 1.4	18.6 ± 1.3
3	19	6 F; 13 M	24.3 ± 2.2	70.7 ± 9.8	176 ± 8.4	26.4 ± 2.0	19.5 ± 1.2

**TABLE 2:** Identified masses and JMs for each session.

Session	*m*_*eq*,*h*_ (kg)	*ϕ*_*h*_ (rad)
1	2.13 ± 0.56	0.27 ± 0.12
2	1.93 ± 0.26	0.21 ± 0.07
3	1.90 ± 0.36	0.23 ± 0.07

Certain participants were involved in the three sessions (*N* = 8 participants). Participants were all naive about the purpose of the experiment. As our goal is to build a control law that can be used by different individuals, most participants from the first two sessions were not the same.

##### ABLE Exoskeleton

**FIGURE 5:**
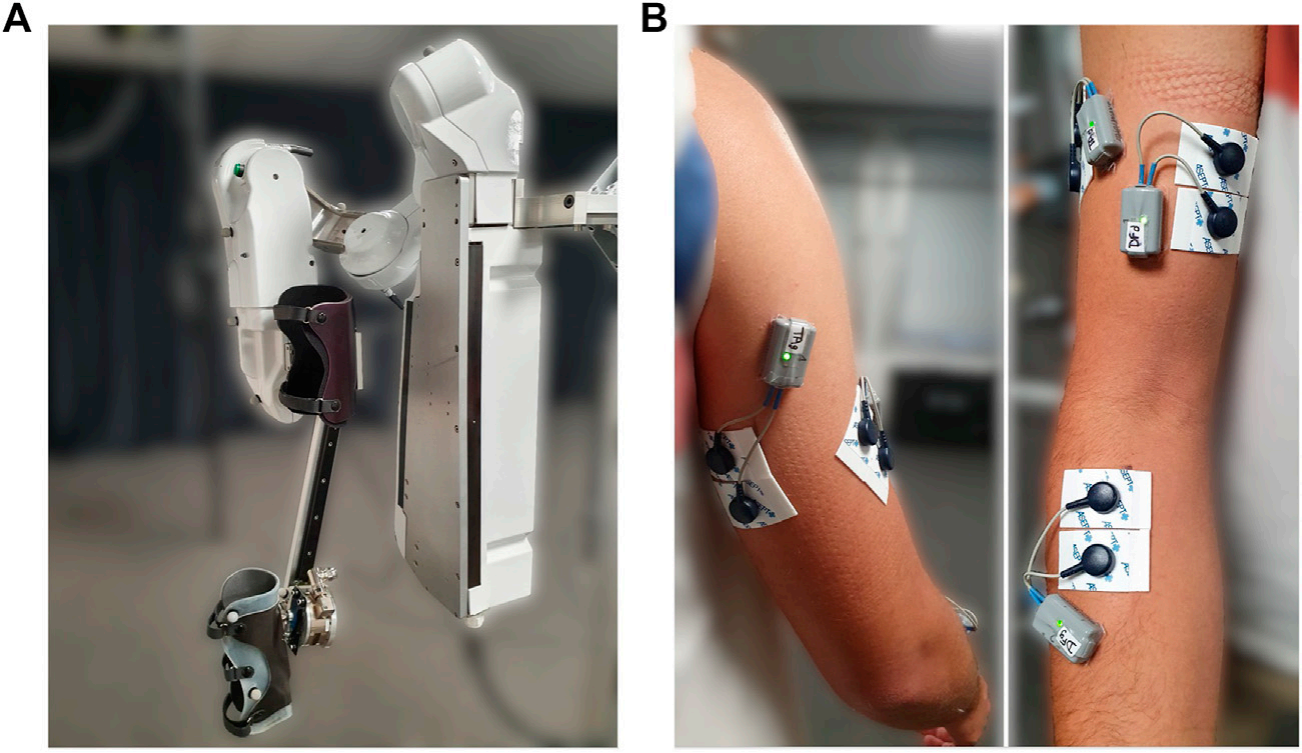
Material and experimental set-up. **(A)** ABLE exoskeleton used in the present study. **(B)** Disposition of EMG sensors (back on left, front on right).

The present study is achieved with an ABLE upper-limb exoskeleton (see Figure 5A) which has four actuated joints and one free slider whose position and velocity cannot be directly monitored.

The first three actuated joints of ABLE correspond to the three degrees of freedom of the human gleno-humeral joint (abduction/adduction, internal/external rotation and flexion/extension) and the last actuated joint correspond to the flexion/extension of the human elbow. The slider is moving on a rail, which forms a prismatic joint, and has been added to prevent the occurrence of hyperstatic forces at the level of the connection between ABLE and the user. Furthermore, a ball joint was added between the slider and the connection to the user with the same purpose and, therefore, provide a more ergonomic interaction (Jarrasse and Morel, 2012). For the same purpose, connections are made of a deformable material to adapt to the shape of the participant’s limb. The forearm connection is also made to impeach wrist movements in order to ensure that movements are only performed by elbow flexion/extension. This robot was designed to be highly backdriveable and compliant in order to maximize the human/exoskeleton symbiosis (Garrec et al., 2008; Garrec, 2010). In addition to classical measures of motor positions obtained in real-time by incremental encoders, a FT sensor (1010 Digital FT, ATI^©^, sample rate: 7 kHz) was added at the level of the connection between ABLE and the user to measure the interaction forces and provide the feedback for WS control laws.

##### Other Materials

Kinematic characteristics of movements were extracted from the robot position data. Participant’s muscular activities were measured with four EMG sensors (Wave Plus wireless EMG system, Cometa). These EMGs were placed on two elbow flexor muscles (brachio-radialis and biceps brachii) and two elbow extensor muscles (triceps brachii long head and lateral head). The EMG sensors are placed according to SENIAM recommendations (Hermens et al., 1999) (see Figure 5B).

##### Motor Task

As previously motivated (see Section 1 and Section 2.1), the task involved only elbow flexions and extensions. Movements consisted in point-to-point reaching in the sagittal plane. Targets are described in Figure 2B as *T*_*up*_, *T*_*bot*_ and *T*_*mid*_. For the participants, these targets are represented by 3 LEDs. The middle LED is the initial target. The two other LEDs are successively illuminated so as to generate movements of an amplitude of 60° centered around a horizontal axis. Participants are asked to move toward the illuminated LED. The LED are illuminated during 1 s to induce a movement duration that stays around 600 ms. Movements are performed under two different conditions, in “transparent” mode and in WS mode (*α* = 1). Between each single point-to-point movement, participants maintained their position during approximately 2 s.

Participants are placed with their shoulder at the intersection of the first three joints of ABLE’s shoulder. They are connected to ABLE at the level of their forearm and arm with large splints to maximize the interaction quality (Jarrasse and Morel, 2012) (see Figure 5A).

In the first session, each participant was asked to perform 7 blocks of 30 movements (15 upward and 15 downward), thereby giving a total of 210 movements per participant. The first block was performed in “transparent” (TR) mode to help the participant to learn the task, then 6 blocks were performed under WS with the BC control law. In the second session, each participant performed the 6 WS blocks with the FFTF control law.

In the final validation experiment, 4 blocks of 30 movements are carried out (15 upward and 15 downward), which yielded a total of 120 movements per participant. The first block was also in “transparent” mode for familiarization and then each block was performed with the BC, FFTF or FFCM control law respectively. These three control laws were randomly assigned to one block. At the end of each block, each participant was asked to point towards each target (*T*_*up*_, *T*_*bot*_ and *T*_*mid*_) and maintain position for five seconds in order to obtain static measurements.

In order to prevent fatigue effects, 3 min pauses were taken between blocks. Note that the performed movements were not particularly demanding due to the presence of a WS (although imperfect) in most conditions.

#### 2.3.2 Data Processing

##### Electromyography

EMG signals were filtered using a band-pass filter (fourth-order Butterworth [20, 450]Hz cut-off frequency), centered and rectified. Signals were normalized by the maximal activity found during the experiment for each subject and each muscle. The envelope of the EMG signal was obtained by applying a low-pass filter (10 Hz cut-off frequency, fifth-order Butterworth) (Potvin and Brown, 2004). The Root mean square (RMS) of the signal was finally computed to measure an amount of activation during one movement and peak activation was computed as the maximum value of the signal for each movement. Possible outliers on peak activation were removed with a threshold at 3 standard deviations. The activity of flexors is computed as the average activity of the brachio-radialis and biceps brachii and the activity of extensors is computed as the average activity of the triceps brachii long head and lateral head.

##### Robot Data

Angular positions of the fourth axis of the robot were recorded by means of internal encoders and angular velocity was estimated by numerical derivation of these positions. The movements were first grossly segmented in the middle of the 2 s waiting time between the lighting of the diodes (as described in Section 2.3.1). The start and end of the movement were then defined using a threshold fixed at 5% of the peak of velocity of the considered movement. Interaction forces were recorded by means of the FT sensor to assess the validity of our compensation. A low-pass filter (5 Hz cut-off frequency, Butter-worth) was applied on robot angular velocity. Acceleration of the fourth axis was then obtained by numerical differentiation of the filtered velocity for offline computations. The “real” interaction torque at the participants’ elbow *τ*_*i*_ was computed as presented in Equation 13,

$$\tau_{i}=lF$$ (13)

where *l* is the distance between the robot elbow and the slider estimated manually at the beginning of the experiment, *F* is the interaction force measured on the *z*-axis (i.e., the normal) of the FT sensor. The distance *l* varies during the experiment but these variations are neglected as they are of small magnitude when compared with the initial distance and have little impact on the weight compensation torque. Theoretical *F* and *τ*_*i*_ were also computed using the model presented in Eq. 7. In all our experiments we fully compensated weight (*α* = 1), therefore the condition *τ*_*i*_ > 0 Nm should be respected.

##### Statistical Analysis

The inter-individuals data were first compared via repeated measures ANOVA with a correction of Greenhouse-Geisser. If statistical differences are observed, data sets are compared using pairwise *t* − test comparisons with a Bonferroni correction as *post-hoc* tests. All statistical analyses are conducted with the Pingouin package (Vallat, 2018). All significance levels are fixed at *p* < 0.05.

## 3 Results

### 3.1 Impact of JM Consideration and Identified Human Arm Parameters

In this subsection, we compare the identification errors for the models with and without JM and report the identified human arm parameters in each case to validate our approach.

**FIGURE 6:**
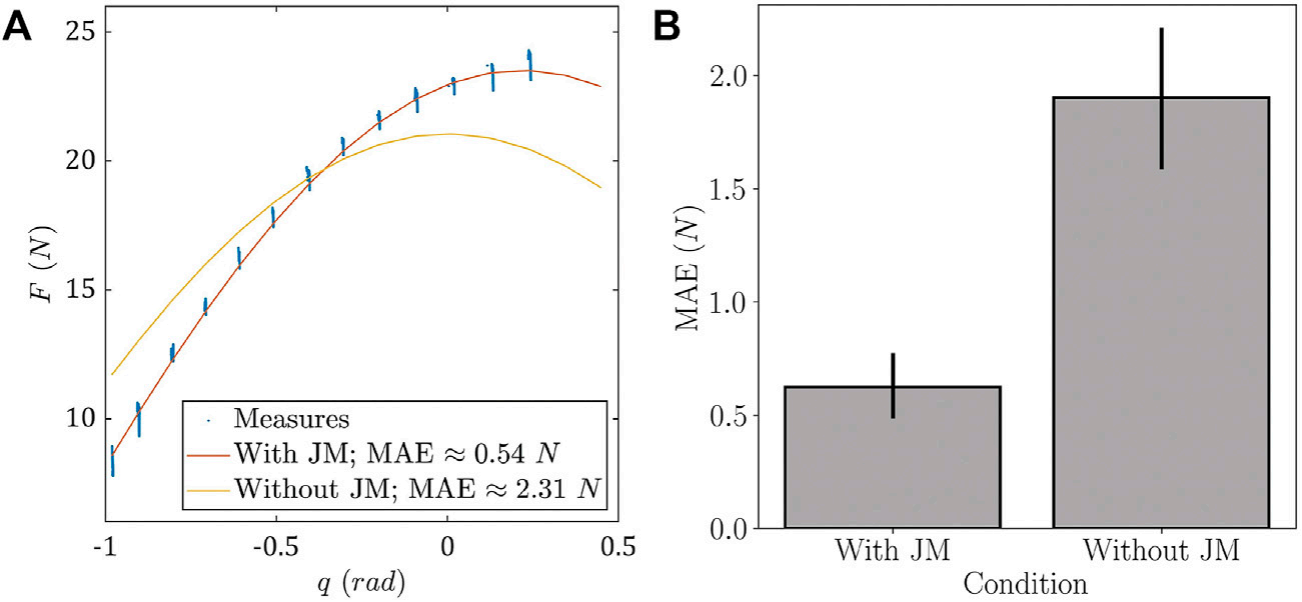
Effects of JM on model behavior and MAEs. **(A)** Example of differences in model identification for a representative participant. **(B)** Boxplots representing MAEs repartition with and without JM.

The results of identification errors are given in Figure 6. The obtained error values are 1.9 ± 0.68 N for the condition without JM (i.e., *ϕ_h_* ≜ 0) and 0.62 ± 0.28 N for the condition introducing JM.

An example of the differences between the two identified models is given in Figure 6A. This example clearly shows that considering JM corrects a substantial amount of error. The arm parameters identified in this example were *m*_*eq*,*h*_ ≈ 2.45 kg; *ϕ* ≈ 0.217 rad with JM and *m*_*eq*,*h*_ ≈ 2.14 kg without. The identification MAEs across participants are displayed for both approaches in Figure 6B, showing the distribution of identification errors both with and without JM through boxplots. The pairwise *t*-test returned a significant difference between the two sets (*T*-value: −7.18, *p* = 3.91 × 10^–7^). Mean and standard deviation of the MAEs show a net decrease in mean error and in error variability. Indeed, the mean error is approximately divided by a factor 3 and the standard deviation is approximately divided by a factor 2.4.

The mean identified *m*_*eq*,*h*_ and *ϕ*_*h*_ and their standard deviations for all participants are *m*_*eq*,*h*_ = 1.93 ± 0.26 kg and *ϕ*_*h*_ = 0.21 ± 0.07 rad.

These results demonstrate the prominent role of JM in achieving a correct human segment mass identification, which is a necessary prerequisite towards an accurate WS. Furthermore, the estimation of the position of the user in the exoskeleton is a data that could be used in other situations such as tasks based on assistive control laws.

As the WS model has now been defined and validated, the next step is the validation of the WS control law designed in Section 2.3.1. This validation is presented in Section 3.2.

### 3.2 Comparison of the Performance of Control Laws

#### 3.2.1 Comparison of Control Laws Static Performances

**FIGURE 7:**
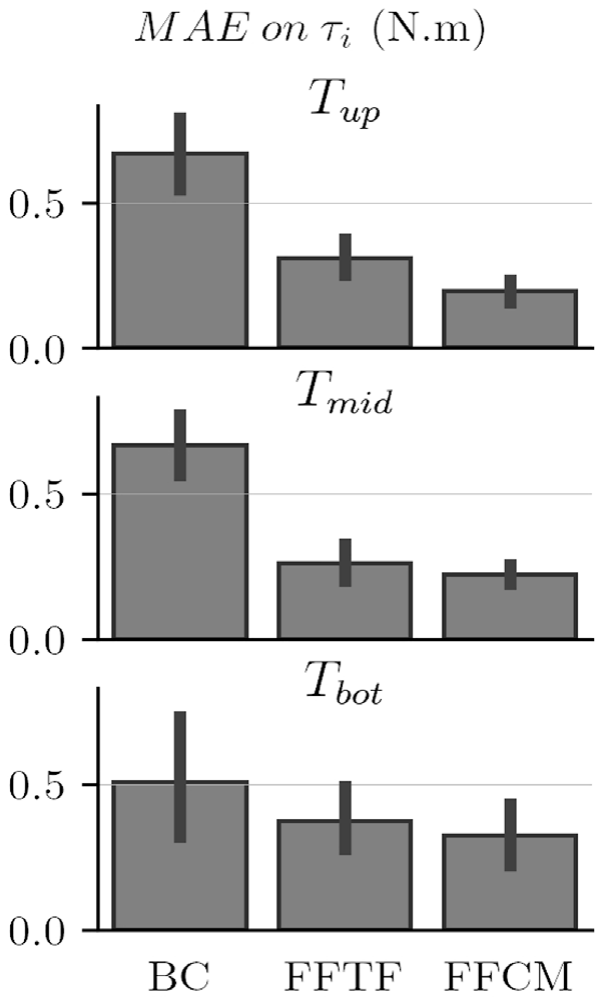
Comparison of the control laws’ performances under static conditions.

An important feature of WS control laws is the ability to remain static when the user is relaxed. Therefore the measurement of errors between the identified model and the measured efforts in static conditions is necessary to quantify the overall quality of these control laws. In the present study, these errors were measured on 5-s pointing on each of the three targets (*T*_*up*_, *T*_*bot*_ and *T*_*mid*_). The obtained results are given in Figure 7.

The results depicted in Figure 7 show a net decrease of the torque-level MAEs by using the FFTF and FFCM control laws. The FFCM control law seems to induce a second reduction of the static MAEs overall. It also seems that this control law induces a reduction in MAE variability for *T*_*mid*_ and *T*_*up*_. Both the FFTF and FFCM control laws induce a net decrease in MAE variability when compared with the BC control law.

The repeated measures ANOVA on control laws and targets returns significant differences between conditions (*F*_2,36_ = 36.7, *p* = 0.3 × 10^–5^, *η* ^2^ = 0.67). No statistical differences are observed between targets. The interaction between targets and control laws is not significant either. Post-hoc treatments show that FFTF and FFCM are significantly better than BC overall (*p* = 1.1 × 10^–4^ and *p* = 0.7 × 10^–5^ respectively). Furthermore, post-hoc treatments show that FFCM offers a significantly better performance than FFTF (*p* = 2.5 × 10^–3^) in terms of MAE on the three targets.

#### 3.2.2 Tracking Performance

**FIGURE 8:**
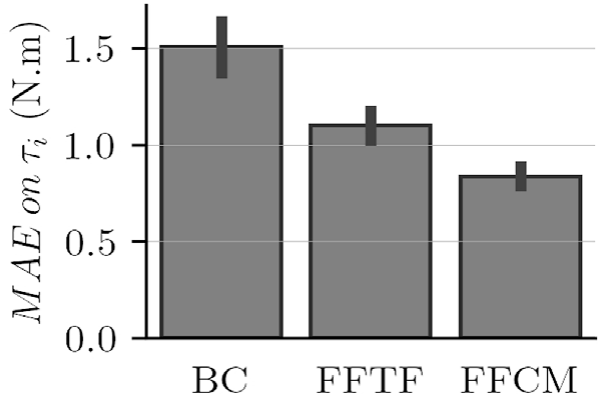
Comparison of the tracking performance each control law in terms of MAE on *τ*_*i*_ for each experimental session.

**FIGURE 9:**
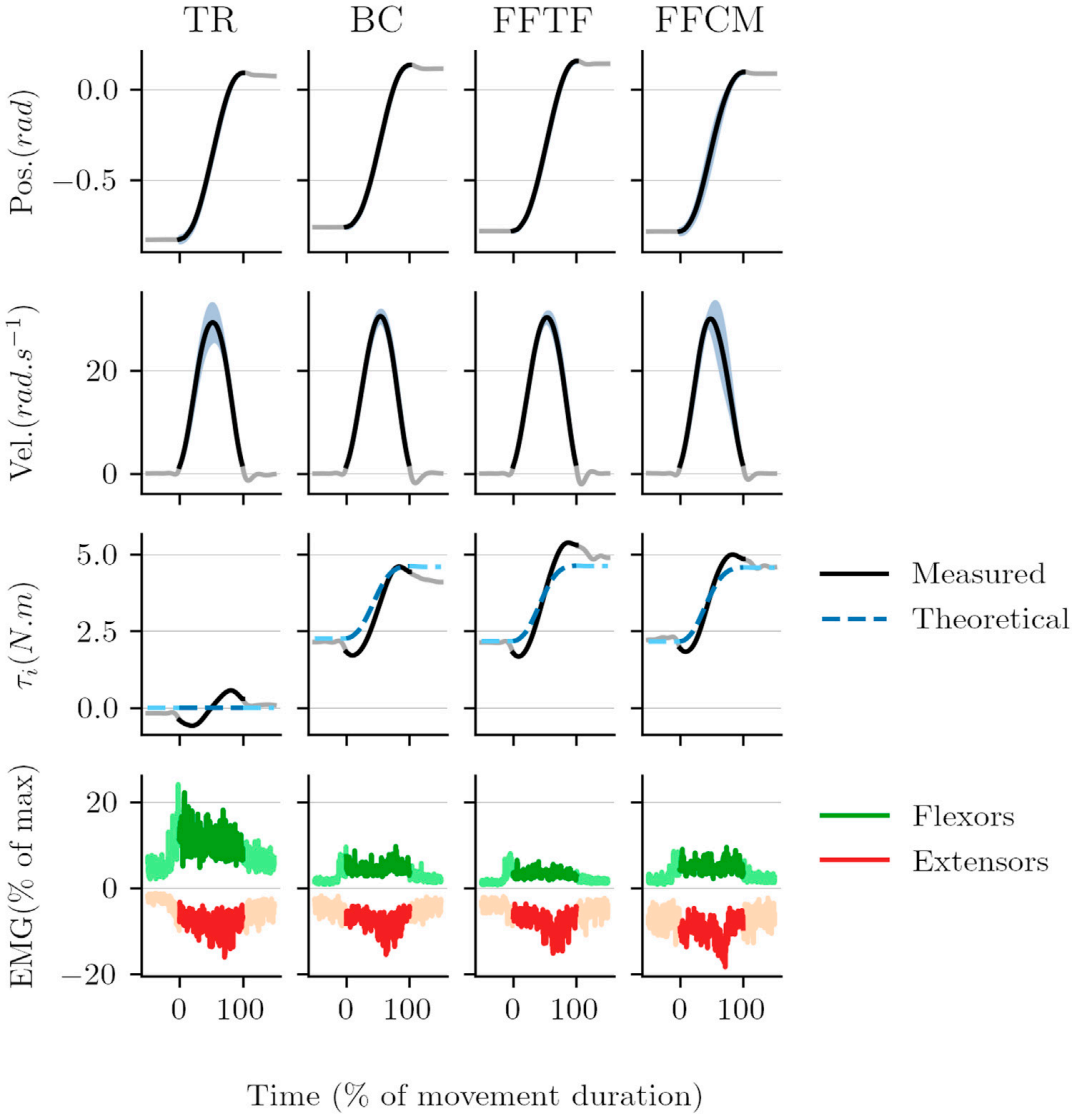
Trajectories, torques, and muscular activation averaged for one participant for upward movements. EMG normalized peak activations are reduced when compared to Figure 11 because of the averaging on multiple movements.

One objective of the present study was to design an accurate WS control law in terms of weight model tracking. Therefore, the best control law must minimize the differences between the predicted interaction force described in Section 2.1 and the measured interaction force *F*. The mean results in terms of model tracking for each control law are visible in Figure 8 and in the third row of Figure 9. Movement traces averaged for one participant are reported for upward movements in Figure 9.

The quantification of the performance of each control law in terms of weight model tracking (i.e., MAE repartition during the whole blocks) is presented in Figure 8. Overall, the FFCM control law performs better (lower average value and with a smaller standard deviation than other laws). This figure reveals a substantial reduction of the MAEs with the addition of each feedforward term. A decrease of approximately 27% of the mean MAE when adding the feedforward term based on the error relative to the theoretical force. The addition of the term based on the error relative to the angular position results in a decrease of approximately 24% of the mean error when comparing FFTF and FFCM and of approximately 44% when comparing BC and FFCM. The variability of the mean MAE also shows a substantial decrease when adding the feedforward terms. These results are promising considering that the aim of the present study is to build a law suitable for the entire population.

The ANOVA analysis returns a significant difference between the different laws (*p* = 2.49 × 10^–11^, *F* = 54.5). The paired *t* − tests conducted on these MAEs all reveal significant improvements in terms of tracking performances: BC vs. FFTF returns *p* = 9.3 × 10^–5^, *T* = 5.61, BC vs. FFCM returns *p* = 5.54 × 10^–8^, *T* = 9.88 and FFTF vs. FFCM returns *p* = 2.85 × 10^–4^, *T* = 5.07.

Eventually, the performance in terms of MAE under dynamic and static conditions is proven to increase with both the FFTF and FFCM control laws, which supports their validity and shows the relevance of their design method a posteriori. Nevertheless, the question of the effects of these control laws on human movement and physiological behavior is still unanswered. This is why these effects are the main focus of Section 3.3.

### 3.3 Effects on Human Trajectories and Muscle Activity

#### 3.3.1 Trajectories

The movement profiles obtained with the three WS control laws were all globally similar to the transparent condition with bell-shaped velocity profiles (see Figure 9 for details on upward movements). Furthermore, Figure 9 does not seem to suggest that there is more overshoot with WS control laws than in transparent mode. This is confirmed by a repeated measures ANOVA on the first sub-movement amplitude that showed no significant difference between the conditions (*F*_3,54_ = 0.446, *p* = 0.721, *η* ^2^ = 0.0242) This suggests that participants successfully adapted their motor planning and correction to WS control laws.

Regarding the basic kinematic features of the movements, we report above the movement duration, peak velocity and peak acceleration in each condition. These averaged values are reported in Table 3. These values were computed on all the movements (pooling both upward and downward movements).

**TABLE 3:** Means and standard deviations of standard descriptors of human movement.

	PV (rad/s)	PA (rad/s^2^)	D (s)
TR	29.1 ± 6.8	157.4 ± 56.2	0.64 ± 0.14
BC	28.8 ± 6.5	155.8 ± 52.3	0.64 ± 0.17
FFTF	30.9 ± 6.9	169.7 ± 60.8	0.61 ± 0.15
FFCM	29.8 ± 6.4	165.3 ± 56.5	0.61 ± 0.12

PV stands for peak velocity, PA stands for peak acceleration and D stands for duration.

Mean values do not show any clear difference between the control laws on these parameters. Peak velocities, peak accelerations and movement duration were similar for all control laws. This was confirmed by repeated measures ANOVA which showed no significant difference between all the control laws for these parameters.

#### 3.3.2 Muscle Activity

As for the analysis of the performance of control laws in Section 3.2, data of EMG sensors were analyzed under two situations: during static positions maintained at the end of the blocks and during dynamic movements. For the latter situation, we focused on the muscular activity during the acceleration phase, which reflects the effort provided by the user to launch the movement and is related to the obtained peak acceleration.

**FIGURE 10:**
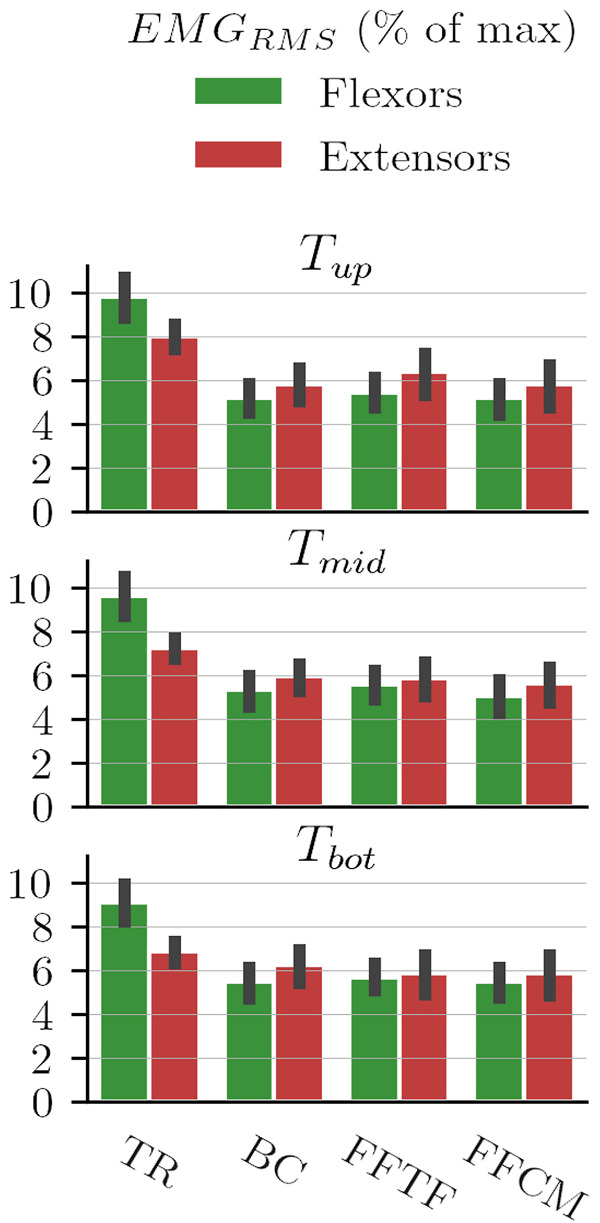
RMS of EMG signals during static position maintenance at the three targets.

The mean results in terms of RMS of EMG signals at the three targets during the static part of the blocks are illustrated by Figure 10.

These results suggest that there is a significant reduction in flexor muscle activity while maintaining static positions. This is confirmed by the repeated measures ANOVA conducted on these data that reports significant differences between conditions (*F*_3,54_ = 553.14, *p* = 2.82 × 10^–28^, *η* ^2^ = 0.97). Post-hoc analyses show that the three WS laws (BC, FFTF and FFCM) induce significantly reduced flexors activity by the participant when compared to the TR condition (*p* = 2.2 × 10^–5^, *p* = 2.1 × 10^–5^ and *p* = 9.5 × 10^–5^ respectively). No statistical differences are observed for extensors activity during post-hoc treatments even though the ANOVA reported a slightly significant main effect (*F*_3,54_ = 3.73, *p* = 0.046, *η* ^2^ = 0.17. This result suggests that differences might exist. Nevertheless, these differences might be due to intra-individual differences during the experimentation as the “transparent” block was always the first block. As expected, WS reduces flexors activity while maintaining static positions across the whole movement range and mostly leaves extensors activity unchanged (in agreement with the fact that these muscles are not critical to counteract gravitational loads in this posture).

**FIGURE 11:**
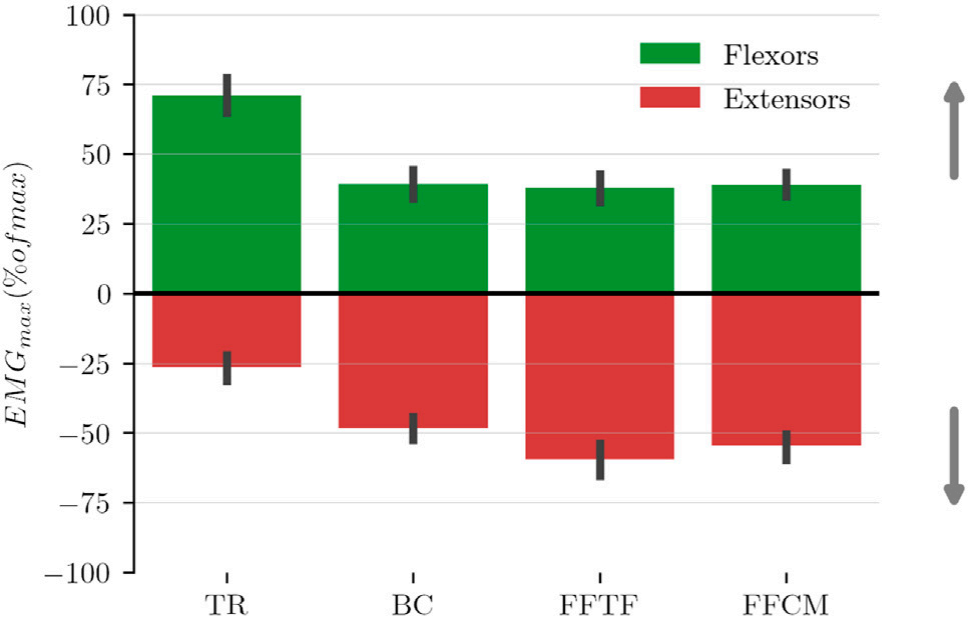
Mean normalized peak of muscular activity of the agonist muscles estimated during the movement acceleration phase. Flexors are agonist for upward movements (depicted with positive values) and extensors are agonist for downward movements (depicted with negative values).

The second step of the analysis was to quantify the dynamic effects of the three WS laws in the sagittal plane. Therefore, the normalized peak of muscular activity of the initial burst of the agonist muscle is a relevant index as it is linked to the effort planned by the participants. The results obtained for upward and downward movements are illustrated in Figure 11.

These mean results demonstrate a decrease in flexors activity to initiate upward movements with the WS control laws, which is coherent with the expected impact of WS control laws. The results also demonstrate an increase in extensor activity to initiate downward movements (which is also coherent with the theoretical effect of WS which prevents participants from taking advantage of weight to move downward). These findings are confirmed by the results of a repeated measures ANOVA that shows a significant difference in flexor activity between conditions (*F*_3_ = 39.82, *p* = 3.52 × 10^–8^, *η* ^2^ = 0.69). Post-hoc analyses report significant differences between the TR and WS conditions for flexors during upward movements (*p* = 1.01 × 10^–7^, *p* = 4.49 × 10^–5^ and *p* = 4.99 × 10^–8^ for BC, FFTF and FFCM respectively). Another repeated measures ANOVA confirms a significant difference in extensors activity between conditions (*F*_3_ = 41.52, *p* = 4.73 × 10^–14^, *η* ^2^ = 0.70). Post-hoc analyses report significant differences between the TR and WS conditions for extensors during downwards movements (*p* = 5.44 × 10^–7^, *p* = 3.25 × 10^–9^ and *p* = 1.46 × 10^–5^ for BC, FFTF and FFCM respectively). No statistical differences are observed between the WS control laws in terms of peak activity for both flexors during upward movements, and extensors during downward movements. As expected WS reduces significantly the activity of flexors during upward movements but increases the activity of extensors activity during downward movements.

## 4 Discussion

### 4.1 Weight Model

The present study introduced a new weight model for WS considering JM between the participant and the exoskeleton. These misalignments have proven to be an important factor to correctly describe the weight of a human segment from the robot’s viewpoint. Even though certain exoskeletons can be adjusted to the user to minimize JM (Kong and Jeon, 2006; Hwang and Jeon, 2015), they cannot be completely canceled (Jarrasse and Morel, 2012) and, therefore, should not be ignored.

### 4.2 Control Laws

The present study tested different control strategies to implement an accurate active WS while moving into the sagittal plane where gravitational torques vary. Our results showed the effectiveness of WS both in static and dynamic situations, although we noticed opposite effects on flexors and extensors at movement initiation, as expected (Gaveau et al., 2019). Other studies have focused on the influence of WS on EMG activity and showed an increase in the number of muscular synergies by looking at ensemble EMG patterns (F. Beer et al., 2007; Prange et al., 2009a; Prange et al., 2009b). Here we obtained clear improvements in the quality of WS in terms of tracking performance for laws including a feedforward term, although the impact on human muscle activity was only visible in statics in our data. Other studies had already described a weight compensation strategy based on a feedforward model-based control (Just et al., 2017; Just et al., 2020). Nevertheless, the performance of this control was not quantified in those works, and the present study suggests that classical approaches might not be sufficient to achieve an accurate WS. As a consequence, feedforward terms learned from error measurements during experiments can improve the accuracy of the impedance controller. These feedforward terms are currently limited to linear models as they are simple to implement and to track for the controller.

The main conclusion that can be drawn is that the use of simple feedforward models based on error measurement is a viable solution for the design of effective WS control laws. Indeed, it has been shown that, even for control laws designed from population-based data (and not personalized), the tracking errors can be significantly reduced compared to classic PI control methods. Furthermore, this design method can be individualised easily if needed, which should lead to even better performance. As it can be seen in Figures 3B, 9, the defined linear models cannot capture, and therefore compensate, errors due to inertial effects and time delay in the feedback control law. The compensation of these errors could provide an important improvement towards more homogeneous active WS.

### 4.3 Effects on Human Movement

Previous studies provided evidence for an increase in work-space size and overall motor performance in stroke patients with WS (Prange et al., 2006; Ellis et al., 2009; Just et al., 2020) but also for comparable movement performances in terms of velocity and trajectory (Prange et al., 2009b,a). In the present study, basic kinematic parameters were also comparable between the TR condition and all the WS control laws, thereby supporting a relative stability of the overall motion kinematics. Therefore, although WS was quantitatively improved using feedforward terms, its impact on human movement data was not obvious. More accurate analyses focusing on the adaptation process or on finer movement parameters could allow to find more subtle effects. For instance, the adaptation of specific movement characteristics such as velocity profile asymmetries, which are well known to be influenced by the ambient gravity field in the motor control literature (Gentili et al., 2007; Gaveau et al., 2016; Bastide et al., 2018), could vary for different control laws. Also, more advanced analyses of ensemble muscle patterns could be applied to detect more complex changes in muscle activities (Chiovetto et al., 2013). The findings of the present study in terms of EMG adaptation are coherent with previous studies that described a decrease in elbow flexors activity when wearing an exoskeleton in WS mode or using a passive device to compensate for weight (F. Beer et al., 2007; Prange et al., 2009a; Prange et al., 2009b; Coscia et al., 2014). The present study also provides data on the increase in elbow extensor activity during movements in a vertical plane when using WS. Future control laws could implement a WS control that activates and deactivates so that patients and workers could exploit gravity to start a downward movement or to brake an upward movement (Gaveau et al., 2019). The present results also suggest that, reducing tracking errors had no significant impact on static and dynamic EMG signals even though variations are observed on average. This is probably due to the variability and noise of EMG sensors that do not allow to observe any clear difference with light interaction force differences. Indeed, if interaction force measures are different (which is the case) but kinematic measures are equivalent (which is also the case), then the differences can only be compensated by muscles activation.

## 5 Conclusion

In the present study, the importance of JM to accurately compensate for the weight of human segments by means of an active exoskeleton was demonstrated and a general model with an associated identification procedure was given. Then, three WS control laws, designed on the basis of experiments, were introduced and tested. The adopted methodology led to significant improvements in model tracking performances and to a control law suitable for the whole tested population. This methodology may thus be suitable to design a fully personalized WS control law for certain rehabilitation or industrial applications. The effects on human movements are comparable to those obtained in previous studies. Furthermore, the measurements of muscular activities suggest that an adaptive WS, which activates only when it helps to reduce muscular effort of antigravity muscles, could be more suitable for certain applications than a complete WS control mode.

## Data Availability

The raw data supporting the conclusions of this article will be made available by the authors, without undue reservation.
