# Supplementary material for: Human Weight Compensation With a Backdrivable Upper-Limb Exoskeleton: Identification and Control
Source: Front Bioeng Biotechnol. 2022 Jan 13;9:796864. doi: 10.3389/fbioe.2021.796864 (PMC8793740; doi:10.3389/fbioe.2021.796864)
Supplement: Supplementary file 1 [file DataSheet1.pdf]

APPENDICES

1 GENERAL WEIGHT MODEL

1.1 Model definition

In the present section, a general model characterizing JM and their influence on weight expression for one revolute robot link and its associated human segment is described. This model could be applied to multiple limbs by adding misalignments and angular parameters. As demonstrated in (Just et al., 2020), it is more efficient to compensate weight based on a vertical projection of the measured efforts (in an absolute frame) with a FT sensor placed at the level of the interaction, thus not using pre-established anthropometric tables to build the weight model. Therefore, the three translations between the human and the robot joints do not have any impact in terms of weight compensation, as long as the human-exoskeleton connection is compliant. Indeed, if the connections are not compliant enough, residual forces due to the hyperstatic nature of the physical connection between the robot and the human may appear and bias the weight model (Jarrasse and Morel, 2012).

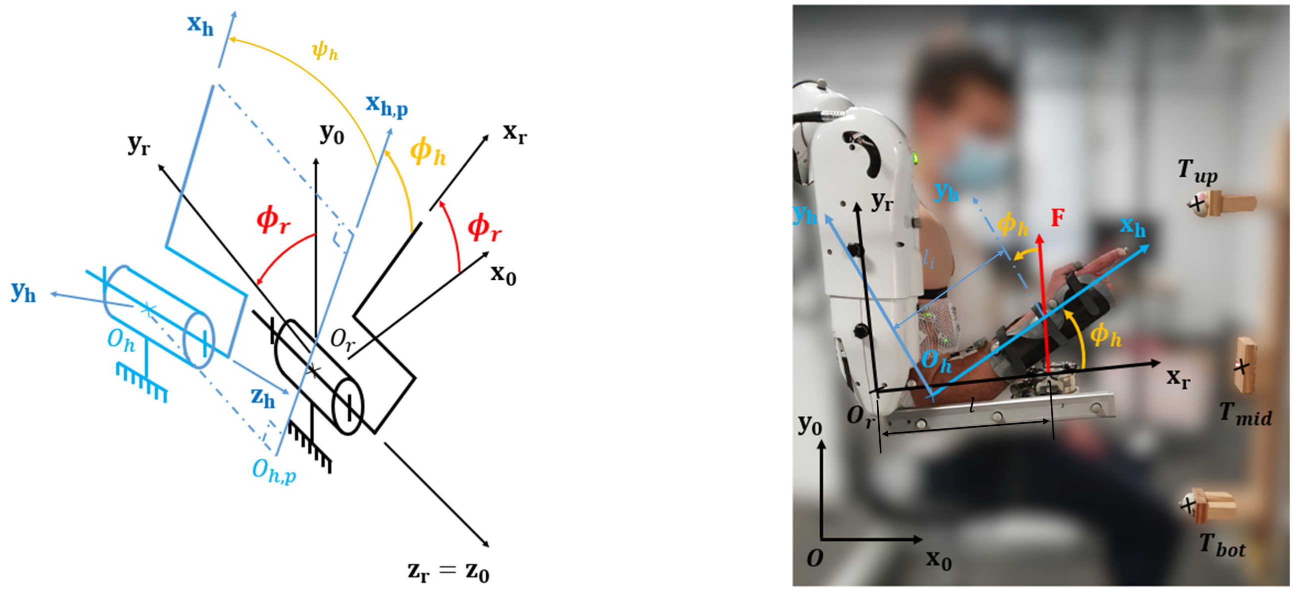

A **B**

Figure 1. Misalignments between human and exoskeleton. **A** General situation. **B** Sagittal plane case study.

The dimension of the orientation problem can be reduced by choosing a proper reference frame taking advantage of the fact that only the orientation of a limb in a vertical plane has an impact on the resulting weight compensation to apply. Indeed, the three elementary rotations involved in the orientation of the human segment and the three ones involved in the orientation of the robot link can be reduced to two rotations if the objective is to compensate weight. To achieve this reduction, the reference frame can be defined with a vertical $\mathbf{y_0}$ axis inducing the other components as described in Equation (1),

$$\begin{cases} \mathbf{z_0} = \dfrac{\mathbf{x_r} \times \mathbf{y_0}}{\| \mathbf{x_r} \times \mathbf{y_0} \|} \\ \\ \mathbf{x_0} = \mathbf{y_0} \times \mathbf{z_0} \end{cases} \tag{1}$$

where $\mathbf{x_0}$ and $\mathbf{z_0}$ are the two other world frame vectors and $\mathbf{x_r}$ is the vector supporting the robot link as described in Figure 1A. The reference frame origin can then be defined as $O = O_r$, where O_r is the origin of the robot link frame. This frame containing the robot link in a vertical plane $(O, \mathbf{x_0}, \mathbf{y_0})$ can always be defined, without any assumption regarding the robot limb orientation. In this frame the orientation of the robot link is only characterized by one rotation as described in Equation (2),

$$\mathbf{x_r} = R_{r0}(\mathbf{z_0}, \phi_r)\mathbf{x_0} \tag{2}$$

where $R_{r0}(\mathbf{z_0}, \phi_r)$ is the rotation of angle ϕ_r around $\mathbf{z_0}$ characterizing the orientation of the robot link in the previously defined vertical plane. This rotation is always known as it only depends on the robot's angular positions and its geometry. In the rest of the present paper, all rotations will be noted $R_{ab}(\mathbf{v}, \theta)$, where R_{ab} is the rotation from frame b to frame a, $\mathbf{v}$ is the vector supporting rotation axis and θ is the angle of rotation. As a result, the robot frame can be defined as described in Equation (3),

$$\begin{cases} \mathbf{y_r} = R_{r0}(\mathbf{z_0}, \phi_r)\mathbf{y_0} \\ \mathbf{z_r} = R_{r0}(\mathbf{z_0}, \phi_r)\mathbf{z_0} = \mathbf{z_0} \end{cases} \tag{3}$$

In this robot link frame, the orientation of the vector supporting the human segment $\mathbf{x_h}$ defined in Figure 1A is defined by two successive rotations. A first rotation to define $\mathbf{y_h} = \mathbf{y_{h,p}}$ and an intermediate $\mathbf{x_h}$ (called $\mathbf{x_{h,p}}$ as defined in Figure 1A), followed by a rotation to define $\mathbf{x_h}$ as described in Equation (4),

$$\begin{cases} \mathbf{y_{h,p}} = R_{hr,1}\left(\mathbf{z_0}, \phi_h(\phi_r)\right)\mathbf{y_r} \\ \mathbf{x_{h,p}} = R_{hr,1}\left(\mathbf{z_0}, \phi_h(\phi_r)\right)\mathbf{x_r} \\ \mathbf{y_h} = \mathbf{y_{h,p}} \\ \mathbf{x_h} = R_{hr,2}\left(\mathbf{y_{h,p}}, \psi_h(\phi_r)\right)\mathbf{x_{h,p}} \end{cases} \tag{4}$$

where $\mathbf{x_h}$ and $\mathbf{x_r}$ are the vectors supporting the human segment and the robot link, respectively. The two human orientation parameters might not be independent from the robot link orientation, therefore they are defined as functions of ϕ_r. The projection of the so-defined $\mathbf{x_h}$ in the plane $(O, \mathbf{x_0}, \mathbf{y_0})$ reduces the orientation of the human segment to the single $\phi_h(\phi_r)$ rotation. This projection is possible because the rotation $R_{hr,2}\left(\mathbf{y_{h,p}}\psi_h(\phi_r)\right)$ does not affect the weight measured in the robot link frame as it does not impact the human segment orientation in the previously defined vertical plane. Eventually, the weight of the human segment can be characterized in the same vertical plane as described in Equation (5),

$$\mathbf{W_h^h} = \mathbf{W_h^{h,p}} = R_{h0}(\mathbf{z_0}, \phi_r + \phi_h(\phi_r))^T \mathbf{W_h^0} \tag{5}$$

where $\mathbf{W_h^h}$ and $\mathbf{W_h^0}$ are the human segment weight expressed in the human and in the world frame, respectively. The rotation ϕ_r is already defined and $\phi_h(\phi_r)$ is the previously defined angle of rotation between the robot and the human segment around $\mathbf{z_r}$. The projected human segment is represented by the

axis $(O_{h,p}, \mathbf{x_{h,p}})$ on Figure 1A. The expression of the weight in the human frame is necessary to compute the gravitational torque at the level of the joint and, therefore, to compute the weight compensation to be applied by the robot. All rotation operators are detailed in appropriate basis in Appendix 1.2.

1.2 Rotation matrices definition

The rotation between the robot link and the world frame expressed in the world frame is presented in Equation (6),

$$R_{r0}(\mathbf{z_0}, \phi_r) = \begin{pmatrix} \cos(\phi_r) & -\sin(\phi_r) & 0 \\ \sin(\phi_r) & \cos(\phi_r) & 0 \\ 0 & 0 & 1 \end{pmatrix} \tag{6}$$

The rotation between the robot link and the projected human segment expressed in the world frame is presented in Equation (7),

$$R_{hr,1}(\mathbf{z_0}, \phi_h(\phi_r)) = \begin{pmatrix} \cos(\phi_h(\phi_r)) & -\sin(\phi_h(\phi_r)) & 0 \\ \sin(\phi_h(\phi_r)) & \cos(\phi_h(\phi_r)) & 0 \\ 0 & 0 & 1 \end{pmatrix} \tag{7}$$

The rotation between the projected human segment and the human segment expressed in the projected human segment frame is presented in Equation (7),

$$R_{hr,2}(\mathbf{y_{h,p}}, \psi_h(\phi_r)) = \begin{pmatrix} \cos(\psi_h(\phi_r)) & 0 & \sin(\psi_h(\phi_r)) \\ 0 & 1 & 0 \\ -\sin(\psi_h(\phi_r)) & 0 & \cos(\psi_h(\phi_r)) \end{pmatrix} \tag{8}$$

The rotation between the world frame and the projected human segment expressed in the world frame is presented in Equation (7),

$$R_{h0}(\mathbf{z_0}, \phi_r + \phi_h(\phi_r)) = \begin{pmatrix} \cos(\phi_r + \phi_h(\phi_r)) & -\sin(\phi_r + \phi_h(\phi_r)) & 0 \\ \sin(\phi_r + \phi_h(\phi_r)) & \cos(\phi_r + \phi_h(\phi_r)) & 0 \\ 0 & 0 & 1 \end{pmatrix} \tag{9}$$

REFERENCES

Jarrasse, N. and Morel, G. (2012). Connecting a Human Limb to an Exoskeleton. IEEE Transactions on Robotics 28, 697–709. doi:10.1109/TRO.2011.2178151

Just, F., Özhan Özen, Tortora, S., Klamroth-Marganska, V., Riener, R., and Rauter, G. (2020). Human arm weight compensation in rehabilitation robotics: efficacy of three distinct methods. Journal of NeuroEngineering and Rehabilitation 17. doi:10.1186/s12984-020-0644-3
